# CT comparison of the nasal airway anterior and posterior to the piriform aperture in patients with and without nasal obstruction

**DOI:** 10.1186/s13005-024-00420-6

**Published:** 2024-03-27

**Authors:** Helen Heppt, Gerlig Widmann, Felix Riechelmann, Annette Runge, Herbert Riechelmann, Aris I. Giotakis

**Affiliations:** 1 Department of Otorhinolaryngology, Medical University of Innsbruck, Anichstrasse 35, Innsbruck, 6020, +435050423141 Austria; 2 Department of Radiology, Medical University of Innsbruck, Innsbruck, Austria; 3 Department of Orthopaedics and Traumatology, Medical University of Innsbruck, Innsbruck, Austria

**Keywords:** Computed tomography, Nasal septum, Nasal obstruction, Inferior turbinate, Respiratory airflow, Case-control studies

## Abstract

**Background:**

Nasal airway stenosis may lie anterior and/or posterior to the piriform aperture. We intended to compare the nasal airway anterior and posterior to the piriform aperture in patients with and without nasal obstruction.

**Methods:**

Segmented computed tomography cross-sectional areas of the nasal airway anterior (CT-CSA_ant_) and posterior to the piriform aperture (at the level of the head of the inferior turbinate; CT-CSA_post_) were compared between patients with nasal obstruction (cases) and trauma controls. CT-CSA were approximately perpendicular to the direction of the nasal airflow. Anterior to the piriform aperture, they were tilted about 30^o^, 60^o^ and 90^o^ to the nasal floor. Posterior to the piriform aperture, they were tilted about 50^o^, 80^o^ and 100^o^ to the nasal floor. In cases, we examined the Pearson’s correlation of active anterior rhinomanometry with CT-CSA_ant_ and CT-CSA_post_.

**Results:**

Narrow and bilateral CT-CSA_post_ were similarly large between 56 cases and 56 controls (all *p* > 0.2). On the contrary, narrow and bilateral CT-CSA_ant_ were significantly smaller in cases than in controls (all *p* < 0.001). The ratio of the size of CT-CSA_ant−30_ to that of CT-CSA_post−80_ was significantly lower in cases (median: 0.84; lower to upper quartile: 0.55–1.13) than in controls (1.0; 0.88–1.16; Mann-Whitney U test; *p* = 0.006). Bilateral CT-CSA_ant_ correlated significantly with total inspiratory flow (all *p* < 0.026) in contrast to bilateral CT-CSA_post_ (all *p* > 0.056).

**Conclusions:**

The nasal airway anterior to the piriform aperture was smaller in patients with nasal obstruction due to skeletal nasal stenosis than that in controls. On the contrary, the nasal airway posterior to the piriform aperture was similarly large between patients with and without nasal obstruction. Furthermore, in patients with nasal obstruction, the anterior nasal airway was narrower compared to that located posterior to it. On the contrary, control patients’ anterior nasal airway was as large as the posterior one.

## Background

Skeletal nasal stenosis is one of the major causes of chronic nasal obstruction. Stenoses may lie anterior and/or posterior to the piriform aperture. The nose anterior to the piriform aperture consists of bone and cartilage [1]. In the anterior nose, deviation of cartilages contributes to nasal obstruction. On the contrary, also bony and mucosal structures, e.g., nasal turbinates, contribute to nasal obstruction in the part of the nose located posterior to the piriform aperture.

The effect of the part of the nose located anterior to the piriform aperture and that located posterior to it on nasal obstruction have not been sufficiently compared. Acoustic rhinometry could aid to this comparison. However, the correlation of acoustic rhinometry with the perception of nasal obstruction is uncertain [2, 3].

Computed tomography (CT) may assist this comparison. CT has a serious disadvantage. It cannot evaluate the condition of the nasal mucosa, which is another major cause of chronic nasal obstruction. However, it has several advantages. It is verifiable, reproducible, not-examiner dependent, easily available, less error-prone than functional rhinometry procedures, without adapter or nozzle related tissue distortion and is associated with ultra-low radiation exposure [4].

In their CT study, Cho and coauthors compared the effect of the anterior and the posterior nose on nasal obstruction, and concluded that they are both related to nasal obstruction. However, the defined anterior nose did not reflect the part of the nose located anterior to the piriform aperture, since it included parts of the inferior and middle turbinate. Furthermore, they investigated only subjects with nasal obstruction [5].

A further advantage of CT is that it can be used in hospital-based case-control studies. A recent study compared CT scans of patients scheduled for nasal surgical procedure due to chronic nasal obstruction (cases) to CT scans of patients with trauma without known clinically relevant nasal obstruction (controls). This setting allowed for significant observations, such as larger nasal floor asymmetry [6], more asymmetric anterior nasal cavities and narrower anterior nasal cavities as a whole in patients with nasal obstruction [7].

With this study, we intended to exploit this setting to compare the anterior nose with the part of the nose that included parts of the inferior turbinate. We measured cross-sectional areas of the nasal airway located anterior to the piriform aperture and posterior to it and were perpendicular to the direction of the nasal airflow, by using reproducible bony landmarks (CT-CSA), in CT scans of patients with clinically relevant nasal obstruction and hospital-based controls.

## Methods

### Study design and population

This was a retrospective hospital-based cross-sectional study. Adult subjects who underwent surgery for chronic nasal obstruction at the University Department of Otorhinolaryngology, Head and Neck Surgery, between January 2017 and December 2020, with a preoperative cone beam CT-scan were eligible (cases). Of these, we used the SPSS random sample routine to identify a sex-balanced random sample. Adult subjects presenting to the Department of Orthopaedics and Traumatology for evaluation and management of serious trauma unrelated to the head and face in the same period served as controls. In subjects serving as controls, multi-slice CT of the head and neck was already available due to routine workup [8]. Subjects were excluded, if nasal cavity or sinus opacification, facial or cephalic dysmorphic syndromes, or facial bone trauma were present. The study protocol was approved by the local ethics committee (1261/2019).

### Computed tomography

The cone beam CT protocol (KaVo 3D eXam, KaVo, Biberach, Germany) used a slice thickness of 0.3 mm, voxel size 0.3 × 0.3 × 0.3 mm, and matrix 536 × 536. The multi-slice CT protocol (Discovery CT750 HD, GE Healthcare, Vienna, Austria) used a slice thickness of 0.6 mm, voxel size 0.625 × 0.391 × 0.391 mm, and matrix 512 × 512 [7]. The target registration error does not differ significantly between cone beam CT and multi-slice CT [9]. This indicates a similar accuracy in both modalities. During the CT-scan, no specific instructions were given to the subjects to breathe through the nose or mouth.

The software Syngo-share-view (Siemens Healthcare Diagnostics GmbH, Vienna, Austria) was used to visualize the DICOM data sets and to carry out the measurements, with default settings for the window and level (window width 3200 and level 600). The multi-window display, using multiplane reconstructions, allowed for simultaneous visualization of axial, sagittal and coronal planes.

### CT-planes

CT-CSA were measured separately for the right and left nose in mm^2^. We assessed the CT-CSA of the part of the nose located anterior to the piriform aperture and of that located posterior to it, at six planes at different angles to the nasal floor, which were defined in a midsagittal plane.

**Fig. 1:**
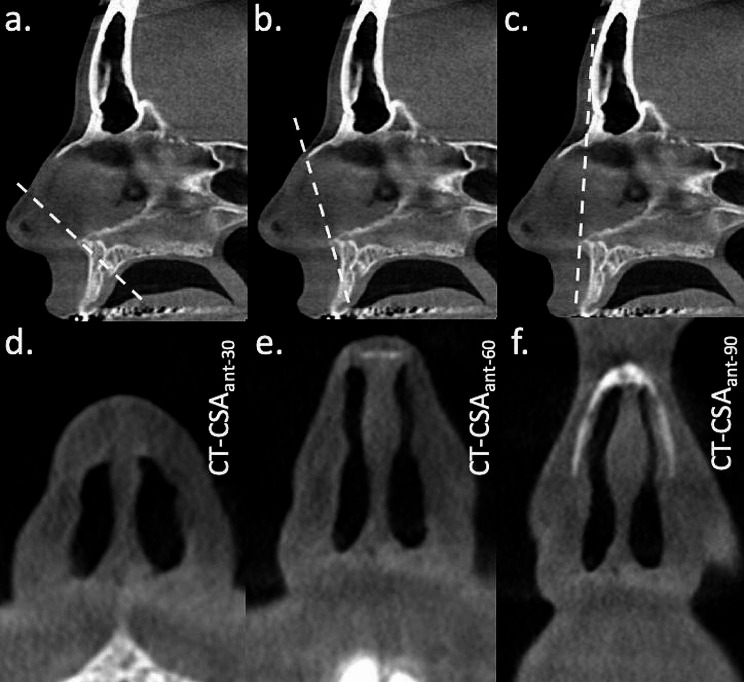
Identification of three planes approximately perpendicular to the curved airflow in the anterior nose in a mid-sagittal section. The white dashed line indicates the selected oblique planes using the anterior nasal spine as a pivot point to the **(a)** posterior edge of the inferior ostium of the incisive canal, **(b)** anterior edge of the intranasal suture and **(c)** most ventral part of the frontal bone. The corresponding cross-sectional areas are shown below: **(d)** CT-CSA_ant−30_, **(e)** CT-CSA_ant−60_, and **(f)** CT-CSA_ant−90_. Note the absence of mucosal structures

The anterior nasal spine was used as the pivot point for determination of all three planes of the part of the nose located anterior to the piriform aperture. These were titled about 30^o^, 60^o^ and 90^o^ to the nasal floor (CT-CSA_ant−30_, CT-CSA_ant−60_ and CT-CSA_ant−90_, respectively). Anterior planes were defined by the anterior nasal spine and the posterior edge of the inferior ostium of the incisive canal (CT-CSA_ant−30_), the anterior edge of the intranasal suture (CT-CSA_ant−60_) and the most ventral part of the frontal bone (CT-CSA_ant−90_; Fig. 1).

**Fig. 2:**
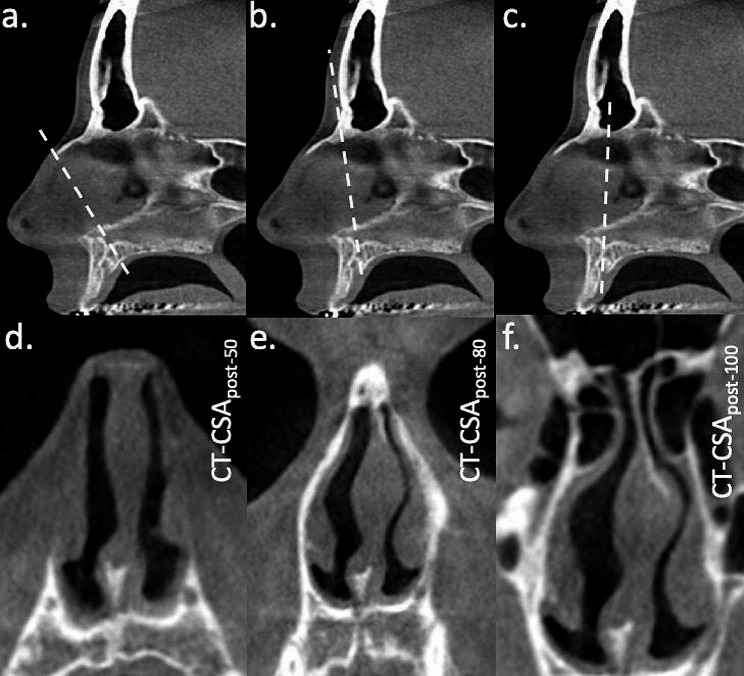
Identification of three planes approximately perpendicular to the curved airflow in the posterior nose in a mid-sagittal section. The white dashed line indicates the selected oblique planes using the anterior edge of the superior ostium of the incisive canal as a pivot point to the **(a)** anterior edge of the intranasal suture, **(b)** most ventral part of the frontal bone and **(c)** posterior edge of the inferior ostium of the incisive canal. The corresponding cross-sectional areas are shown below: **(d)** CT-CSA_post−50_, **(e)** CT-CSA_post−80_ and **(f)** CT-CSA_post−100_. Note the presence of mucosal structures

The anterior edge of the superior ostium of the incisive canal was used as the pivot point for determination of all three planes of the part of the nose located posterior to the piriform aperture. These were titled about 50^o^, 80^o^ and 100^o^ to the nasal floor (CT-CSA_post−50_, CT-CSA_post−80_ and CT-CSA_post−100_, respectively). Posterior planes were defined by the anterior edge of the superior ostium of the incisive canal and the anterior edge of the intranasal suture (CT-CSA_post−50_), the most ventral part of the frontal bone (CT-CSA_post−80_) and the posterior edge of the inferior ostium of the incisive canal (CT-CSA_post−100_; Fig. 2).

### Segmentation

**Fig. 3:**
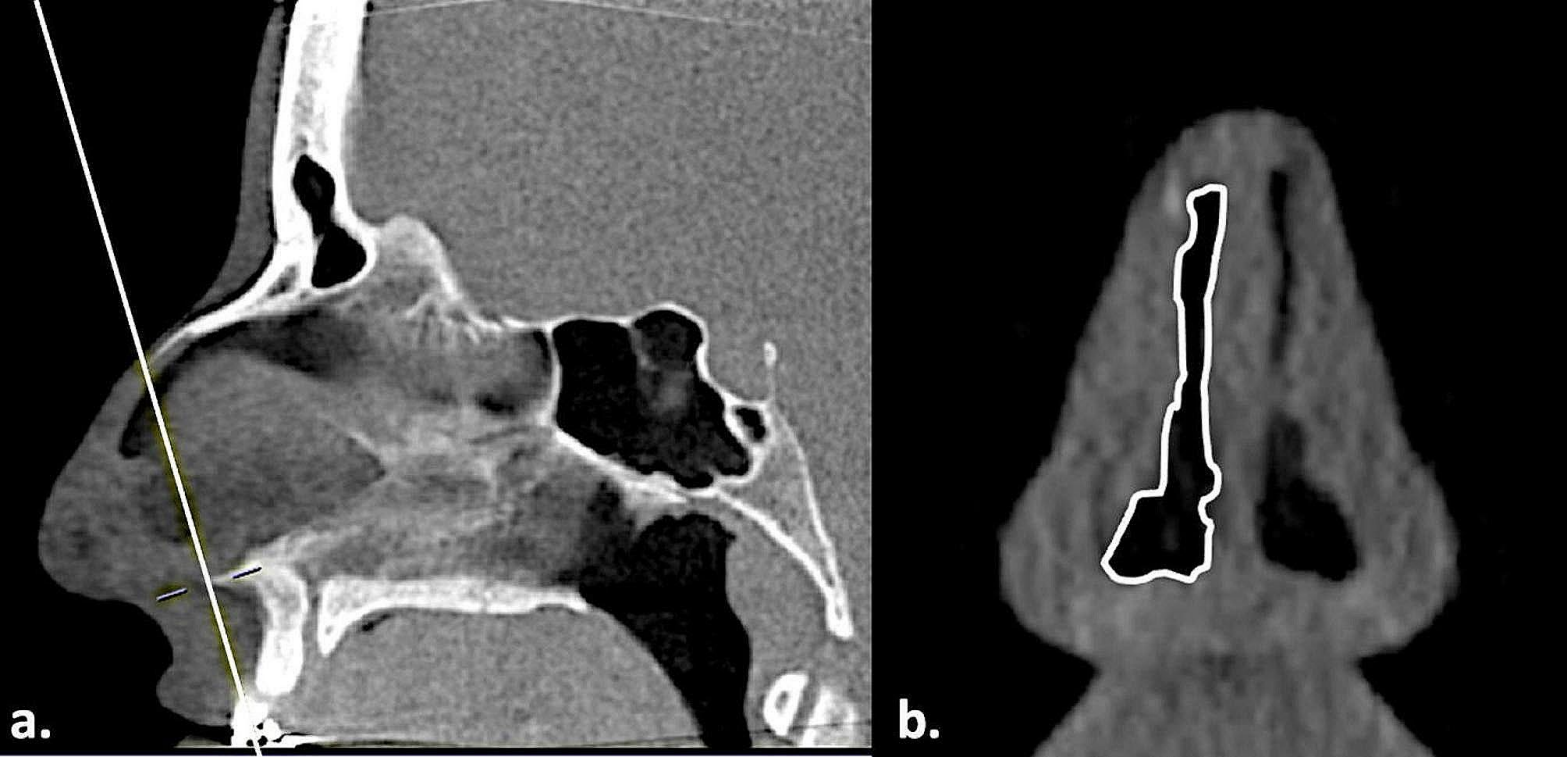
Segmentation of the CT-CSA. (**a**) Design of the CT-CSA_ant−60_ in a 40-year-old man, by drawing a line (white line) between the anterior nasal spine and the anterior edge of the intranasal suture in the midsagittal plane. (**b**) Outlining the nasal airway by drawing a line (white continuous line) at the border between the black space and the grey area

Segmentation of the CT-CSA was reproducibly carried out manually, by an individual investigator, in the multi window display. Only one investigator carried out the measurements due to limited personnel resources. The investigator was not blinded to the assignment of CT to cases or controls. Firstly, adjustments were made in the axial plane. Here, the caudal nasal septum was set as the midline. Further adjustments were made in the sagittal plane. Here, the landmarks for the desired plane were set (Fig. 3a). These steps resulted in the depiction of the desired oblique plane in the coronal plane. To measure the desired oblique plane, the drawing polygon function was used. The border between the black space of the nasal airway and the grey area of the surrounding tissue was outlined with the mouse (Fig. 3b). This resulted in the depiction of the airway’s surface in mm^2^ by the program. In patients with interruption of the airway’s air space due to soft tissue collapse, the air-containing areas were separately measured and added up [7].

### Active anterior rhinomanometry

We used the Otopront Rhino-Sys system (Otopront, Hohenstein, Germany). No subject had used nasal xylomethazoline spray on the examination day before the measurement. Prior to the examination, each subject waited 15 min to adapt to the indoor climate [10]. Active anterior rhinomanometry (three breathing cycles on average) were performed before and 10 min after decongestion with three puffs (approximately 180 µl) per side of nasal xylometazoline spray 0.05%. Inspiration flow (ml/s) and inspiration resistance (sPa/ml) at 150 Pa were automatically displayed for the left nose, the right nose and bilaterally, and before and after decongestion.

### Data analysis

Data were analyzed using the SPSS 26.0 statistic package (SPSS Inc., Illinois, USA). Count data were tabulated, for metric data means, standard deviations and 95% confidence intervals (CI) were calculated. All angles are in degrees. Normality of distribution of variables was tested with the Shapiro-Wilk test. Pearson’s correlation coefficient was used for two continuous parameters. Independent samples T-test or Mann-Whitney U-test were used for comparison between cases and controls. All analyses comparing cases and controls were adjusted for age. We examined the correlation of the CT-CSA_ant−30_ with the CT-CSA_post−80_, and the ratio of the CT-CSA_ant−30_ to the CT-CSA_post−80_ in order to investigate the association between the parts of the nose located anterior and posterior to the piriform aperture. The following three parameters were derived from the raw data [7].

#### Absolute side differences of CT-CSA in CT

The absolute value of the difference of the right from the left CT-CSA in mm^2^ for each of the six planes served as a measure of the asymmetry of the width of the nasal airway in that plane. The side differences provided a measure of the asymmetry of the actual width of the nasal airway and were compared between cases and controls.

#### Bilateral cross-sectional areas in CT

The CT-CSA of the right and left nose were added for each plane, separately. The bilateral CT-CSA served as a measure of the width of the actual nasal airway and were compared between cases and controls. Furthermore, we examined the correlation of bilateral CT-CSA with total inspiratory flow and total inspiratory resistance of active anterior rhinomanometry in cases.

#### Narrow cross-sectional areas in CT

Under the assumption that the narrow sides contribute more to nasal obstruction, the CT-CSA of the anterior and posterior noses on the narrow sides were also used for comparison between cases and controls. The smaller nasal side of both was defined as the narrow one.

### Results and analysis

#### Study population

During the study period, 1005 patients underwent nasal surgical procedure for chronic nasal obstruction. Of them, a sex-balanced random sample of 60 subjects was drawn for pragmatic reasons. Of them, 56 subjects fulfilled the study criteria and were included. Twentynine were women. The median age was 31 years (range: 18–60 years). Septoplasty and functional septorhinoplasty were carried out by 30 and 26 subjects, respectively. An equal sized sample with balanced gender distribution was provided by the Departments of Orthopaedics and Traumatology and of Radiology. These 56 trauma-subjects were used as controls. Of these, 30 were men. Age did not differ significantly between cases and controls (Mann-Whitney U test; *p* = 0.071).

### Absolute side differences of the cross-sectional areas in CT

#### Anterior CT-CSA side differences

**Fig. 4:**
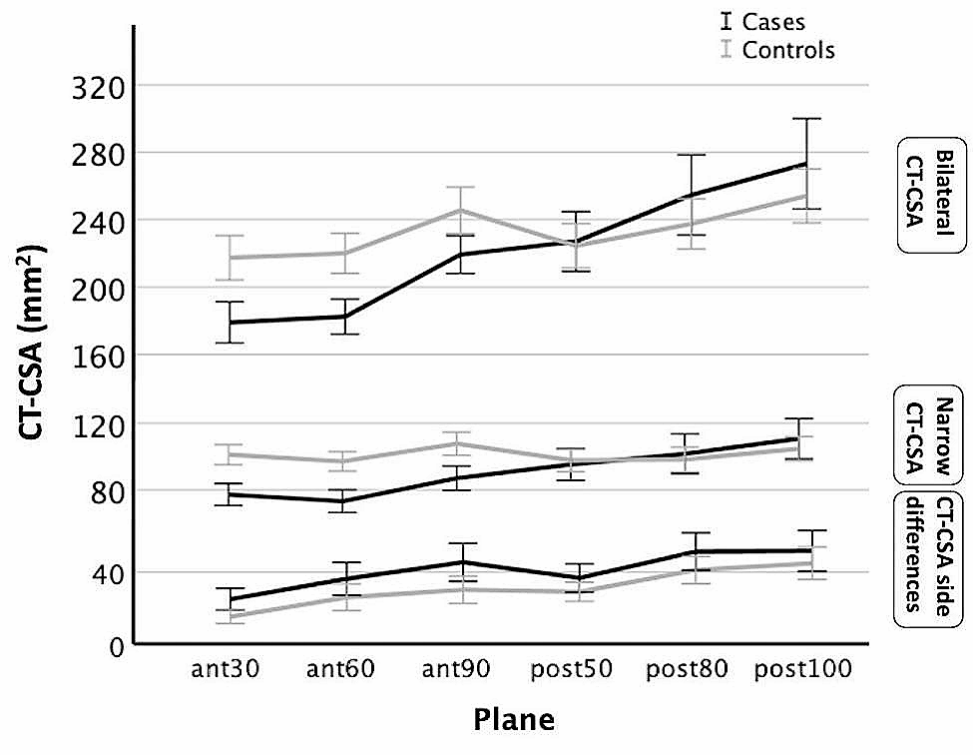
Absolute side difference [abs(right-left); mean ± 95 CI] of the nasal cross-sectional areas, CT-CSA of the total nose (both sides; mean ± 95 CI) and size of CT-CSA on the narrow side of the nose (mean ± 95 CI). In the anterior nose (ant-30, ant-60 and ant-90), note larger side differences in cases than in controls, indicating a greater nasal asymmetry in cases. Side differences in the posterior nose (post-50, post-80 and post-100) were similar between cases and controls. Also, note narrower total nasal airway width of the anterior nose in cases than in controls, but similar width of the total nasal airway between cases and controls in the posterior nose. Lastly, note smaller CT-CSA on the narrow side of the anterior nose in cases than in controls (all *p* < 0.001), but similar size of CT-CSA on the narrow side in the posterior nose between cases and controls. X-Axis: planes; Y-Axis: mean size of CT-CSA in mm^2^

Side differences at the plane of CT-CSA_ant−30_ were on average 9.7±3.6 mm^2^ larger in cases than in controls (*p* = 0.008), at the plane of CT-CSA_ant−60_ they were 10.3±5.9 mm^2^ larger in cases than in controls (*p* = 0.081), and at the plane of CT-CSA_ant−90_ they were 15.3±6.5 mm^2^ larger in cases than in controls (*p* = 0.021; all p Bonferroni-adjusted for multiple comparisons and adjusted for age; Fig. 4).

#### Posterior CT-CSA side differences

Side differences at the plane of CT-CSA_post−50_ were on average 7.7±4.7 mm^2^ smaller in cases than in controls (*p* = 0.11), at the plane of CT-CSA_post−80_ they were 10.3±6.4 mm^2^ smaller in cases than in controls (*p* = 0.11), and at the plane of CT-CSA_post−100_ they were 6.9±7.2 mm^2^ smaller in cases than in controls (*p* > 0.2; all p Bonferroni-adjusted for multiple comparisons and adjusted for age; Fig. 4).

### Bilateral cross-sectional areas in CT (bilateral CT-CSA)

#### Bilateral anterior CT-CSA

Bilateral anterior CT-CSA correlated significantly with age (*p* < 0.009). Bilateral CT-CSA of the anterior nasal airway at the 30^o^ plane were 39.9±8.6 mm^2^ smaller in cases than in controls (*p* < 0.001), at the 60^o^ plane they were 39.1±7.4 mm^2^ smaller in cases than in controls (*p* < 0.001), and at the 90^o^ plane they were 27.9±8.4 mm^2^ smaller in cases than in controls (*p* < 0.001; all p Bonferroni-adjusted for multiple comparisons and adjusted for age; Fig. 4).

#### Bilateral posterior CT-CSA

Bilateral CT-CSA of the posterior nasal airway at the 50^o^ plane were 2.0±11 mm^2^ larger in cases than in controls (*p* > 0.2), at the 80^o^ plane they were 15.8±13.9 mm^2^ larger in cases than in controls (*p* > 0.2), and at the 100^o^ plane they were 17.1±15.3 mm^2^ larger in cases than in controls (*p* > 0.2; all p Bonferroni-adjusted for multiple comparisons and adjusted for age; Fig. 4).

### Narrow cross-sectional areas in CT

Narrow anterior CT-CSA were significantly smaller in cases than in controls (independent samples T-test; all *p* < 0.001; Fig. 4). On the contrary, narrow posterior CT-CSA did not differ significantly between cases and controls (Mann-Whitney U-test; all *p* > 0.2; Fig. 4). The mean value ± standard deviation of CT-CSA_ant−30_ was 77±25 mm^2^ in cases and 101±23 mm^2^ in controls. The median value ± 95% lower and upper bound of confidence interval of CT-CSA_post−80_ was 87 (90;114) mm^2^ in cases and 94 (91;106) mm^2^ in controls (Tables 1 and 2).

**Table 1:** Raw data of nasal airway cross-sectional areas in CT-scans of 56 cases and 56 controls

Parameter	Planes	Subjects with nasal obstruction (Cases)	Controls
Right	Ant-30^1^	85±26 (26–142)	108±28 (66–194)
	Ant-60^1^	85±28 (7-138)	113±30 (64–207)
	Ant-90^1^	104±35 (28–193)	129±35 (61–251)
	Post-50^2^	111 (84;143)	114 (90;140)
	Post-80^2^	117 (86;148)	117 (94;141)
	Post-100^2^	131 (95;168)	114 (96;149)
Left	Ant-30^1^	94±30 (23–151)	110±25 (64–185)
	Ant-60^1^	97±34 (24–184)	107±28 (59–179)
	Ant-90^1^	115±38 (38–203)	117±30 (57–202)
	Post-50^2^	112 (83;135)	111 (86;134)
	Post-80^2^	108 (80;178)	118 (92;134)
	Post-100^2^	122 (82;180)	122 (101;155)

^1^Mean value ± standard deviation (minimum-maximum) in mm^2^

^2^Median value (lower and upper quartile) in mm^2^

**Table 2:** Nasal airway CT cross-sectional areas on the narrow sides in 56 cases and 56 controls

Parameter	Planes	Subjects with nasal obstruction (Cases)	Controls
Narrow	Ant-30^1^	77±25 (64–174)	101±23 (84-116)
	Ant-60^1^	73±26 (7-138)	97±22 (59–149)
	Ant-90^1^	87±27 (28–159)	108±26 (57–187)
	Post-50^2^	89 (65;115)	94 (76;115)
	Post-80^2^	87 (69;132)	94 (92;120)
	Post-100^2^	98 (71;147)	105 (92;120)

^1^Mean value ± standard deviation (minimum-maximum) in mm^2^

^2^Median value (lower and upper quartile) in mm^2^

### Correlation of the bilateral CT-CSA with active anterior rhinomanometry

Active anterior rhinomanometry was available in 30 cases. Bilateral CT-CSA did not correlate significantly with total inspiratory resistance, neither before nor after decongestion (all *p* > 0.11). Similarly, bilateral posterior CT-CSA did not correlate significantly with total inspiratory flow, neither before nor after decongestion (all *p* > 0.056).

Only bilateral anterior CT-CSA correlated significantly with total inspiratory flow. We noted a poor positive correlation between total inspiratory flow and bilateral CT-CSA_ant−90_ before (*r* = 0.53; *p* = 0.003) and after decongestion (*r* = 0.47; *p* = 0.011), and bilateral CT-CSA_ant−60_ after decongestion (*r* = 0.41; *p* = 0.026).

### Association of the CT-CSA located anterior to the piriform aperture with those located posterior to it

#### Correlation

**Fig. 5:**
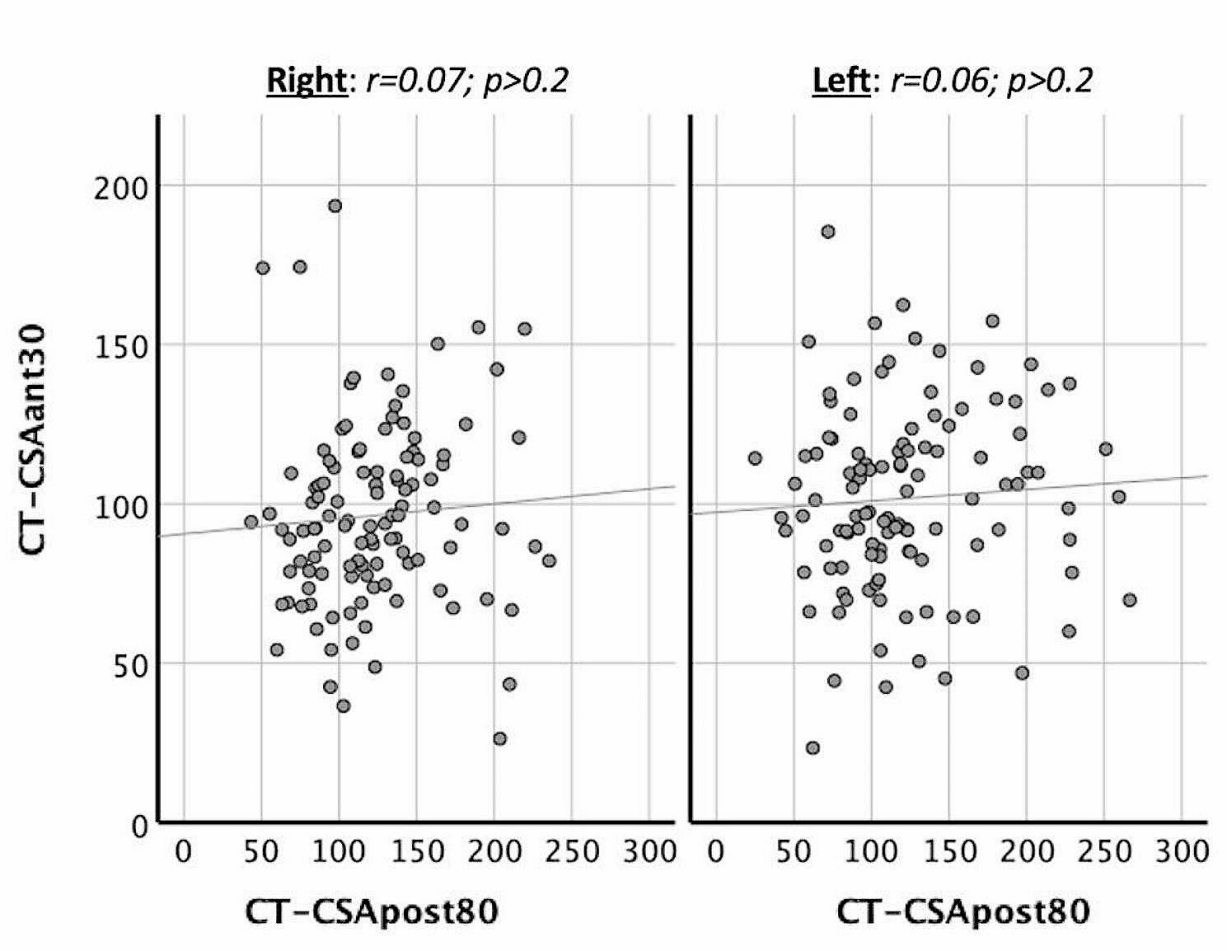
Correlation of the size of the anterior nose (CT-CSA_ant−30_) with the size of the posterior nose (CT-CSA_post−80_) on the right and left nose in all subjects (*n* = 112). The size of the CT-CSA_ant−30_ did not correlate with the size of the CT-CSA_post−80_, neither on the right (*p* > 0.2) nor on the left nose (*p* > 0.2). Y-Axis: size of CT-CSA_ant−30_ in mm^2^; X-Axis: size of CT-CSA_post−80_ in mm^2^

In all 112 subjects, the CT-CSA of the anterior nasal airway (CT-CSA_ant−30_) did not correlate significantly with the CT-CSA of the posterior nasal airway (CT-CSA_post−80_), neither on the right (*r* = 0.07; *p* > 0.2) nor on the left noses (*r* = 0.06; *p* > 0.2; Fig. 5). We noted no significant correlations neither in 56 cases nor in 56 controls.

#### Ratio

**Fig. 6:**
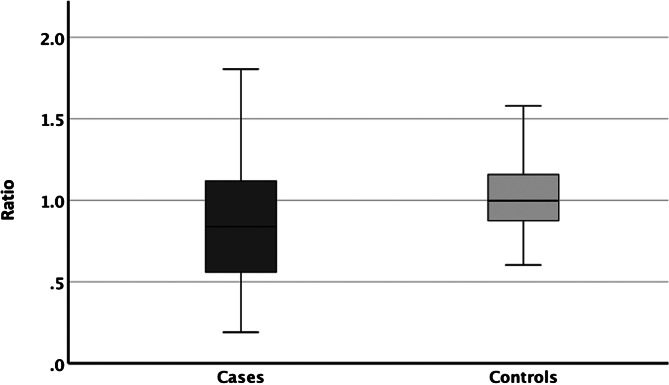
Boxplots of the ratios of the anterior (CT-CSA_ant−30_) to the posterior noses (CT-CSA_post−80_). The latter was significantly lower in cases (median value: 0.84) than in controls (1.00; *p* = 0.006). X-Axis: Subject type; Y-Axis: Ratio (CT-CSA_ant−30_/CT-CSA_post−80_).

The ratio of the anterior to the posterior noses was significantly lower in cases (median: 0.84; lower to upper quartile: 0.55–1.13) than in controls (1.0; 0.88–1.16; Mann-Whitney U test; *p* = 0.006; Fig. 6).

## Discussion

In this retrospective cross-sectional study, we intended to compare the part of the nose located anterior to the piriform aperture with the part of the nose that included the inferior turbinate, slightly posterior to the piriform aperture. A recent study has highlighted the importance of the anterior nasal airway on nasal obstruction [7]. The authors used a hospital-based setting [6], which allowed the comparison of CT cross-sectional areas between a group of subjects with clinically relevant nasal obstruction and a group of subjects without it. These cross-sectional areas were reproducible due to defined bony landmarks, and perpendicular to the direction of the nasal airflow due to exploitation of the anterior nasal spine as a pivot point. This study revealed that anterior nasal cavities were more asymmetric in patients with nasal obstruction and, as a whole, narrower than in patients without clinically relevant nasal obstruction. However, the part of the nose investigated in this study [7] did not include the inferior turbinates.

This motivated us to use the same hospital-based setting to investigate the part of the nose with the inferior turbinate. This reflects the part of the nose located posterior to the piriform aperture. To investigate the inferior turbinate, we selected the part of the nose around the incisive canal. This decision was based on the presence of the inferior turbinates on that plane, and on the presence of the incisive canal, the superior ostium of which was used as the pivot point in the posterior nose (Fig. 2). Furthermore, the bony incisive canal allowed reproducibility of the measurements. Eventually, we titled the anterior nose without inferior turbinates “anterior nose”, and the more posterior part of the nose with inferior turbinates “posterior nose”.

Our results revealed that absolute side differences of the posterior noses, which were considered a measure of asymmetry, were similar in cases and controls. Furthermore, together the right and left CT cross-sectional areas of the posterior noses, which correspond to the total nasal airway width in the posterior nose, were similarly large between cases and controls. Moreover, the narrow sides of the posterior noses were also similarly large between cases and controls (Fig. 4).

These results indicated that the cross-sectional areas of the nasal airway in the posterior nose did not differ significantly in any way (all *p* > 0.2) between patients with nasal obstruction and patients without it. This finding is the opposite to that of the anterior nose, where a larger asymmetry, a narrower total nasal airway width and smaller narrow sides were found in patients with nasal obstruction compared to patients without it. Furthermore, the ratio of the part of the nose located anterior to the piriform aperture with the part of the nose located posterior to it was smaller in patients with clinically relevant nasal obstruction than in controls. These findings implied a more conical-shape opening of the noses of patients with clinically relevant nasal obstruction in contrast to those of controls. There is a paucity of data concerning these findings. Therefore, these may be considered new insights in nasal obstruction caused by skeletal nasal stenosis.

These observations might mislead readers to suppose that only the anterior nose contributes to nasal obstruction and that the posterior nose does not. However, this would be incorrect. Here, we examined the CT cross-sectional areas of the nasal airway and not the whole posterior nose. While the anterior CT cross-sectional areas are usually a direct derivate of the position of the nasal septum, the posterior CT cross-sectional areas depend additionally on the inferior turbinates. These may shape the airway in various ways [11, 12]. One common finding in the posterior nose is a severely deviated nasal septum with contralateral compensatory turbinate hypertrophy. While the deviated nasal septum narrows the airway on one side, the turbinate narrows the airway on the other side [13]. This may lead to similarly large cross-sectional areas on each side of the nose. The position of the nasal septum is an additional parameter that should be examined on CT before nasal surgical procedures [14]. It should be straight enough to allow for unobstructed laminar airflow [15, 16]. If only the CT cross-sectional areas were taken into account during examination of the nose, this could lead to the false impression of unobstructed nasal breathing.

The findings of this study allow additional observations. The anterior noses in total are narrower in patients with nasal obstruction than in patients without it, which is not routinely corrected during septoplasy [7]. Here, we found that the sizes of the CT cross-sectional areas in the posterior nose as a whole, do not differ between patients with nasal obstruction and patients without it. A similar study revealed that the width and height of the piriform aperture, as well as the thickness of the nasal septum do not differ between patients with nasal obstruction and patients without it [6]. It remains unclear to what extent size and/or symmetry of the anterior and posterior nasal airway influence nasal obstruction. The design of this study did not allow for further conclusions.

Our results also revealed that the sizes of the CT cross-sectional areas of the nasal airway in the anterior nose did not correlate with those in the posterior nose, a finding observed in cases as well as in controls (all *p* > 0.2; Fig. 5). This implied that the size of the posterior nose does not relate in any way with the size of the anterior nose. For this comparison, we chose the CT-CSA_ant−30_ and the CT-CSA_post−80_. The CT-CSA_ant−30_ was chosen as the most significant anterior plane for nasal obstruction [7]. CT-CSA_post−80_ had two advantages. It did not overlap with any of the anterior planes and it was closer to the anterior nose than the CT-CSA_post−100_. Moreover, the CT cross-sectional areas of the posterior nasal airway did not correlate with age in contrast to the CT cross-sectional areas of the anterior nasal airway. The latter was in line with recent reports [17, 18].

This study also revealed that total inspiratory flow correlated significantly with bilateral anterior CT-CSA and not with bilateral posterior CT-CSA. This implied that the inspiratory flow increases as the total size of the anterior nasal airway increases. On the contrary, it indicated that the inspiratory flow does not change significantly as the total size of the posterior nasal airway increases. These observations were in line with the findings described so far. We examined the correlation of active anterior rhinomanometry with the bilateral CT-CSA only, because this was the only examined parameter that corresponded to an automatically depicted variable (total flow) of the active anterior rhinomanometry software.

Furthermore, the posterior CT-CSA were more likely to represent the area of the internal nasal valve due to the presence of the head of the inferior turbinate (Fig. 2), compared to the anterior CT-CSA, which would most likely represent the area of the external nasal valve (Fig. 1). The internal nasal valve is considered the narrowest area of the nasal airway. Interestingly, the anterior CT-CSA were narrower compared to the posterior CT-CSA in subjects with nasal obstruction (Table 2). Therefore, we would except the internal nasal valve to be located somewhere between the anterior and posterior CT-CSA.

This study had some limitations. Subjective assessment of nasal breathing with NOSE score [19, 20] would allow for more significant observations. Usually, the first examination used by otorhinolaryngologists to evaluate nasal obstruction is the anterior rhinoscopy. This assesses not only the nasal structure, e.g., septal deviation, but also the condition of the nasal mucosa. While CT may quantify the structural findings of anterior rhinoscopy in a way, it cannot evaluate the condition of the nasal mucosa. The lack of evaluation of the NOSE score in subjects with nasal obstruction was the major limitation of this study.

Objective assessment of nasal patency with acoustic rhinometry [21] was not examined here; this would exceed the resources of this manuscript. The latter applies also for other CT measurements such as the position of the nasal septum [22, 23] or the size of the inferior turbinates [12, 24]. Therefore, the cross-sectional areas examined here did not cover the whole length of the nasal airway comprehensively. Moreover, an increased number of subjects might have unraveled a larger variety of structural problems.

Moreover, hospital-based case-control studies have typical disadvantages [25]. For example, we cannot exclude with certainty that some trauma controls did not suffer from nasal obstruction. However, if nasal obstruction does not increase the risk of trauma, trauma controls should be representative of subjects of the general population [6].

Furthermore, a blinded segmentation would have been preferable. Also, we did not assess the reproducibility of the CT-CSA between different investigators due to limited personnel resources. However, reproducibility of the anterior CT-CSA was expected to be higher than that of the posterior CT-CSA due to the larger amount of mucosa found posteriorly. Random measurements by two different investigators revealed differences in the decimal places.

Lastly, we did not assess the effect of the variations of the nasal resistance, which would mainly affect the posterior CT-CSA due to presence of the inferior turbinates. Nevertheless, not only narrow posterior cross-sectional areas, but also both right and left nasal airway cross-sectional areas, i.e., bilateral posterior CT-CSA, did not differ between cases and controls. The latter, i.e., both right and left nasal cross-sectional areas, if taken together, neutralize the unilateral effect of the nasal resistance’s variations, since the whole nasal cavity, i.e., both right and left, is assessed simultaneously.

On the contrary, this study has significant advantages. The use of CT allowed for hospital-based controls in contrast to other assessment methods of nasal patency. Moreover, CT facilitated the reproducible segmentation of nasal structures due to the multiplanar reconstruction and the bony landmarks. These advantages have been sufficiently discussed in older reports [6, 7].

Future studies may use this hospital-based case-control setting to analyze the data with computational fluid dynamics, and investigate the effect of intended surgical steps in subjects with nasal obstruction. Moreover, it would be interesting to investigate subjects with nasal obstruction and controls with elastometry [26].

## Conclusions

The nasal airway anterior to the piriform aperture was smaller in patients with nasal obstruction due to skeletal nasal stenosis than that in controls. On the contrary, the nasal airway posterior to the piriform aperture was similarly large between patients with and without nasal obstruction. Furthermore, in patients with nasal obstruction, the anterior nasal airway was narrower compared to that located posterior to it. On the contrary, control patients’ anterior nasal airway was as large as the posterior one.

## Data Availability

All data generated or analysed during this study are included in this published article (and its supplementary information files).

## List of abbrevations

CT: computed tomography.
CT-CSA: cross-sectional areas of the nasal airway that were perpendicular to the direction of the nasal airflow by using reproducible bony landmarks.
CT-CSA: _ant−30_: plane defined by the anterior nasal spine and the posterior edge of the inferior ostium of the incisive canal.
CT-CSA: _ant−60_: plane defined by the anterior nasal spine and the anterior edge of the intranasal suture.
CT-CSA: _ant−90_: plane defined by the anterior nasal spine and the most ventral part of the frontal bone.
CT-CSA: _post−50_: plane defined by the anterior edge of the superior ostium of the incisive canal and the anterior edge of the intranasal suture.
CT-CSA: _post−80_: plane defined by the anterior edge of the superior ostium of the incisive canal and the most ventral part of the frontal bone.
CT-CSA: _post−100_: plane defined by the anterior edge of the superior ostium of the incisive canal and the posterior edge of the inferior ostium of the incisive canal.
CI: confidence intervals.
